# *Echinococcus granulosus* cyst fluid inhibits KDM6B-mediated demethylation of trimethylated histone H3 lysine 27 and interleukin-1β production in macrophages

**DOI:** 10.1186/s13071-023-06041-3

**Published:** 2023-11-16

**Authors:** Ruolin Lin, Xiaopeng Wang, Caiya Ni, Chunxue Fu, Chun Yang, Dan Dong, Xiangwei Wu, Xueling Chen, Lianghai Wang, Jun Hou

**Affiliations:** 1 NHC Key Laboratory of Prevention and Treatment of Central Asia High Incidence Diseases, Shihezi University School of Medicine, Shihezi, Xinjiang China; 2 Key Laboratory of Xinjiang Endemic and Ethnic Diseases, Shihezi University School of Medicine, Shihezi, Xinjiang China

**Keywords:** Cystic echinococcosis, NLRP3 inflammasome, KDM6B, Trimethylated histone H3 lysine 27

## Abstract

**Background:**

*Echinococcus granulosus* can manipulate its host's immune response to ensure its own survival. However, the effect of histone modifications on the regulation of the NOD-like receptor protein 3 (NLRP3) inflammasome and downstream interleukin-1β (IL-1β) production in response to the parasite is not fully understood.

**Methods:**

We evaluated IL-1β secretion through enzyme-linked immunosorbent assay and assessed reactive oxygen species levels using the dichlorodihydrofluorescein diacetate probe. Western blotting and quantitative real-time polymerase chain reaction were performed to examine the expression of NLRP3 and IL-1β in mouse peritoneal macrophages and Tohoku Hospital Pediatrics-1 cells, a human macrophage cell line. The presence of trimethylated histone H3 lysine 27 (H3K27me3) modification on NLRP3 and IL-1β promoters was studied by chromatin immunoprecipitation.

**Results:**

Treatment with *E. granulosus* cyst fluid (EgCF) considerably reduced IL-1β secretion in mouse and human macrophages, although reactive oxygen species production increased. EgCF also suppressed the expression of NLRP3 and IL-1β. Mechanistically, EgCF prompted the enrichment of repressive H3K27me3 modification on the promoters of both NLRP3 and IL-1β in macrophages. Notably, the presence of EgCF led to a significant reduction in the expression of the H3K27me3 demethylase KDM6B.

**Conclusions:**

Our study revealed that EgCF inhibits KDM6B expression and H3K27me3 demethylation, resulting in the transcriptional inhibition of NLRP3 and IL-1β. These results provide new insights into the immune evasion mechanisms of *E. granulosus*.

**Graphical Abstract:**

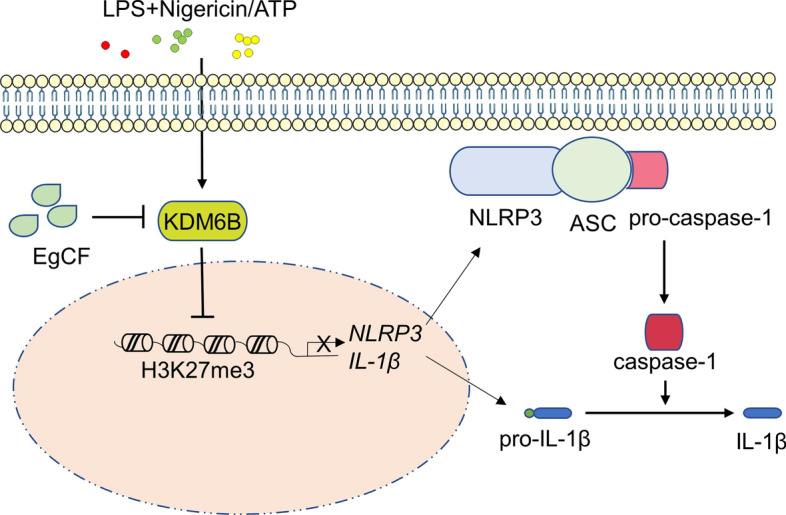

**Supplementary Information:**

The online version contains supplementary material available at 10.1186/s13071-023-06041-3.

## Introduction

Echinococcosis, a zoonotic disease of global prevalence, remains a substantial public health concern [1]. Cystic echinococcosis (CE) and alveolar echinococcosis are zoonotic tapeworm ailments caused by *Echinococcus granulosus* sensu lato and *Echinococcus multilocularis*, respectively [2]. CE is prevalent in western China, Central Asia, South America, the Mediterranean Region, and East Africa, with exposure to domestic ungulates being the primary risk factor [3, 4]. As the parasite has evolved, it has developed efficient escape mechanisms to avoid immune system attacks by its host [5]. *Echinococcus granulosus* is known to suppress innate inflammatory responses to evade inflammatory reactions involving macrophages [5]. Moreover, the parasite can shield itself from the body’s anti-parasitic immune response for extended periods of time with the help of *E. granulosus* cyst fluid (EgCF) [6, 7]. Therefore, further studies on how *E. granulosus* interacts with its host are crucial to developing novel immunotherapies.

The NOD-like receptor protein 3 (NLRP3) inflammasome is essential in regulating the host’s innate immune response. It can recognize various pathogen-associated molecular patterns and damage-associated molecular patterns to maintain the stability of the body’s internal environment [8]. The activation of NLRP3 inflammasome in macrophages follows a two-signal model. The transcription of interleukin-1β (IL-1β) and NLRP3 is increased in response to the first signal, the nuclear factor kappa B (NF-κB) pathway stimulated through Toll-like receptors [9]. The second signal, which includes substances such as nigericin, adenosine 5′-triphosphate (ATP), and reactive oxygen species (ROS), activates the inflammasome. This activation triggers proteolytic conversion from pro-IL-1β to mature IL-1β, which is subsequently released by the cell. In various types of parasitic infections, parasites have been found to inhibit NLRP3 inflammasome, resulting in immune evasion. For instance, the *Leishmania* RNA virus hinders the activation of NLRP3 and the release of IL-1β to facilitate the survival of *Leishmania guyanensis* [10]. Additionally, *Toxoplasma gondii* can evade human neutrophil-mediated host defense by inhibiting NF-κB signaling and preventing the activation of the NLRP3 inflammasome [11]. However, the role of the NLRP3 inflammasome in *E. granulosus* infection requires further research.

KDM6B is an inducible histone demethylase that removes repressive trimethylated histone H3 lysine 27 (H3K27me3) epigenetic marks [12, 13]. It plays a crucial role in the epigenetic regulation of inflammation [14–16]. The expression of KDM6B can be triggered by various inflammatory mediators like lipopolysaccharide (LPS) and TNFα, and it specifically demethylates H3K27me3 to boost the transcription of inflammatory genes [17–20]. However, the function of histone modifications and KDM6B in macrophages during *E. granulosus* sensu lato infection remains poorly understood.

This article presents evidence that EgCF can suppress inflammatory responses by inhibiting KDM6B expression and promoting the repressive H3K27me3 modification, thereby decreasing the transcription of NLRP3 and IL-1β, and ultimately reducing the production of IL-1β.

## Methods

### Cell culture

Mouse peritoneal macrophages were isolated as previously described [21]. Briefly, C57BL/6 mice (6–8 weeks old, 18–22 g body weight) were sacrificed and intraperitoneally injected with Dulbecco’s modified Eagle medium (Gibco), which was supplemented with 10% fetal bovine serum (Gibco) and 1% penicillin–streptomycin (Gibco). The abdomen was massaged for 5 min, and then the contents were withdrawn. The collected peritoneal exudate cells were centrifuged at 300 *g* for 5 min and grown in complete Dulbecco’s modified Eagle medium medium under a 5% CO_2_ atmosphere at 37 ℃ for 24 h. Next, the cells were washed with phosphate-buffered saline (PBS) to remove non-adherent cells. The human monocytic cell line Tohoku Hospital Pediatrics-1 (THP-1) was purchased from Pricella and maintained in RPMI 1640 medium (Gibco) containing 10% fetal bovine serum, 1% penicillin–streptomycin, and 0.05 mM β-mercaptoethanol (Gibco). The THP-1 cells were differentiated into macrophages after treatment with 100 ng/mL of phorbol-12-myristate-13-acetate for 24 h. LPS (L8880; Solarbio) was the first signal to activate the NLRP3 inflammasome [9]. ATP disodium salt hydrate (A2383; Sigma) and nigericin sodium salt (S25116; Yuanye) were used as the second signals for NLRP3 inflammasome activation [22].

### Preparation of *E. granulosus* cyst fluid

Sheep livers infected with *E. granulosus* were purchased from a slaughterhouse in Changji, Xinjiang. After disinfecting the surface of the sheep liver, the contents of the vesicles were extracted and centrifuged at 1000 *g* for 10 min. The supernatant was then filtered through a 0.22-μM filter. The protein concentration of EgCF was quantified using a bicinchoninic acid protein assay kit, and the fluid was stored in a refrigerator at – 80 ℃ for later use.

### Enzyme-linked immunosorbent assays

Cell culture medium was collected and centrifuged at 300 *g* for 10 min, and the supernatant was collected. IL-1β concentration was determined by using a human IL-1β enzyme-linked immunosorbent assay (ELISA) kit (EK101B; Multi Sciences) and mouse IL-1β ELISA Kit (EK201B; Multi Sciences), in accordance with the manufacturer’s instructions, and the optical density at 450 nm (wavelength correction at 570 nm) was measured using a Varioskan LUX Multimode Microplate Reader (Thermo Scientific).

### Detection of intracellular ROS

Intracellular levels of ROS were measured by using the Reactive oxygen species Assay Kit (Nanjing Jiancheng Bioengineering Institute) in strict accordance with the manufacturer’s instructions. An appropriate amount of dichlorodihydrofluorescein diacetate (10 μM) diluted in serum-free medium was added to the cell culture plate and the contents incubated at 37 ℃ for 30 min. The sample was subsequently observed for dichlorofluorescein fluorescence under an inverted fluorescence microscope (Shanghai Guangmi Instrument) at ×200 magnification. Relative fluorescence intensity was measured using ImageJ.

### Western blotting

Proteins were extracted using radioimmunoprecipitation assay lysis buffer, isolated using polyvinyl gel electrophoresis, and transferred onto a polyvinylidene fluoride membrane. The membrane was blocked with a solution of 5% bovine serum albumin and incubated with the following antibodies, as appropriate, overnight at 4℃: NLRP3 (ab263899; Abcam), ASC (no. 67824, no. 13833S; Cell Signaling Technology), caspase-1 (A0964; ABclonal), IL-1β (no. 12242S; Cell Signaling Technology), β-actin (TA-09; ZSGB Biotech), H3K27me3 (no. 9733S; Cell Signaling Technology), H3K9me3 (no. 13969S; Cell Signaling Technology), H3K36me3 (no. 4909S; Cell Signaling Technology), H3 (no. 9751S; Cell Signaling Technology). The membranes were washed 3 times for 10 min with 0.1% Tris buffered saline with Tween 20 then incubated with a horseradish peroxidase-conjugated secondary antibody for 1 h at room temperature. After washing, an enhanced chemiluminescence assay (Biosharp) was used to detect the immune complexes. To ensure consistency, β-actin served as a loading control. Original Western blots are shown in the Additional file 1.

### Quantitative real-time polymerase chain reaction

The total RNA of the macrophages was harvested using a total RNA kit (Omega) and reverse-transcribed into complementary DNA using the RevertAid RT Reverse Transcription Kit (Thermo Scientific). Quantitative real-time polymerase chain reaction (qRT-PCR) was performed in a CFX96 Touch detection system (Bio-Rad) using the UltraSYBR One-Step RT-qPCR Kit (CWBIO). The relative expression levels of the target genes were normalized to β-actin by applying the 2^−ΔΔCt^ method and expressed as fold changes to that of the control group. The primer sequences are listed in Table 1.

**Table 1:** Primer sequences for quantitative real-time polymerase chain reaction and chromatin immunoprecipitation-polymerase chain reaction analysis

Name	Forward (5′ → 3′)	Reverse (5′ → 3′)
*Nlrp3*	ATTACCCGCCCGAGAAAGG	CATGAGTGTGGCTAGATCCAAG
*Il1b*	GAAATGCCACCTTTTGACAGTG	TGGATGCTCTCATCAGGACAG
*Il6*	CTGCAAGAGACTTCCATCCAG	AGTGGTATAGACAGGTCTGTTGG
*Kdm6b*	TGAAGAACGTCAAGTCCATTGTG	TCCCGCTGTACCTGACAGT
*Actb*	GGCTATGCTCTCCCTCACG	GAGCAACATAGCACAGCTTCTCTTT
*NLRP3*	GATCTTCGCTGCGATCAACAG	CGTGCATTATCTGAACCCCAC
*IL1B*	ATGATGGCTTATTACAGTGGCAA	GTCGGAGATTCGTAGCTGGA
*IL6*	GATGGATGCTTCCAATCTGGAT	AGTTCTCCATAGAGAACAACATA
*KDM6B*	TTGGGCAACTGTACGAGTCAG	CCATAGTTCCGTTTGTGCTCAAG
*ACTB*	GAGAAAATCTGGCACCACACC	GGATAGCACAGCCTGGATAGCAA
*NLRP3* promoter	GAGTGCCTAGCCTGTGGAAA	CATCCTTTAATGTCTCCTTGCAAAA
*IL1B* promoter	TCCACTTTTCAGAGTTCACCA	GGGGCAGAGAACATACGGTA
*Nlrp3* promoter	TGGCTTCTTTGAGCCACTCT	AAGCCCCAAAAGCCTAGAAG
*Il1b* promoter	ACCTTTGTTCCGCACATC	GGGATTATTTCCCCCTGG

### Chromatin immunoprecipitation-PCR analysis

Chromatin immunoprecipitation-PCR analysis (ChIP) was performed with an anti-H3K27me3 antibody (no. 9733S; Cell Signaling Technology) and anti-immunoglobulin G as a negative control. In brief, following the steps of the SimpleChIP Enzymatic Chromatin IP Kit (agarose beads, no. 9002; Cell Signaling Technology), cells were cross-linked with 1% formaldehyde for 10 min and then quenched by 10 × glycine for 5 min at room temperature to form DNA–protein cross-links. Next, the samples were sonicated to prepare DNA fragments with a size range of 200–1000 base pairs and then incubated with antibodies at 4 ℃ overnight. After the reversal of the protein-DNA cross-linking, the DNA fragment was purified, and PCR amplification was performed. The primer sequences for the ChIP assay are listed in Table 1.

### Immunofluorescence assay

Cells were grown on glass coverslips and fixed with 4% paraformaldehyde for 30 min. After permeabilization with 0.1% Triton X-100, the cells were washed 3 times with PBS and blocked for 1 h at room temperature with 1% bovine serum albumin. Next, the samples were incubated with the anti-KDM6B antibody (ab169197; Abcam) in PBS with Tween 20 and 1% bovine serum albumin overnight at 4 ℃. After washing the cells 3 times with PBS, they were incubated with rhodamine-conjugated goat anti-rabbit immunoglobulin G (H + L) and secondary antibody (ZF-0511; ZSGB-BIO) for 1 h at room temperature and counterstained with 4′,6-diamidino-2-phenylindole. Finally, the cells were imaged under a fluorescence microscope (Olympus).

### Statistical analysis

All experiments were independently repeated 3 times. The data are shown as mean + SEM. GraphPad Prism version 8.0 software was utilized to perform the statistical analyses. One-way ANOVA followed by Tukey’s post hoc test was applied to assess the differences between multiple groups.

## Results

### EgCF inhibits IL-1β secretion by macrophages

**Fig. 1:**
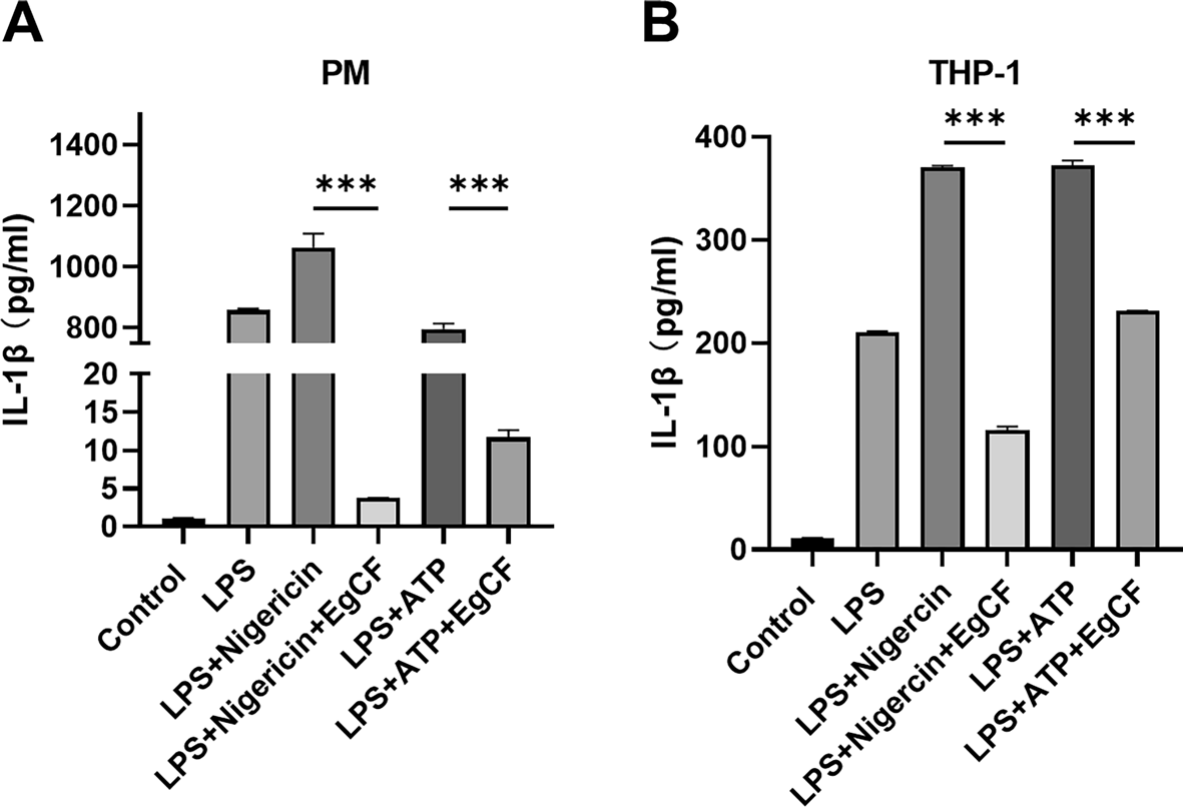
**A**–**B** *Echinococcus granulosus* cyst fluid (*EgCF*) inhibits interleukin-1β (*IL-1β*) secretion triggered by inflammasome inducers in macrophages. Mouse peritoneal macrophages (*PM*) (**A**) and Tohoku Hospital Pediatrics-1 (*THP-1*) cells (**B**) were primed with lipopolysaccharide (*LPS*) (1 μg/mL) in the presence or absence of EgCF (50% v/v) for 4 h and then stimulated with nigericin (10 μM) or adenosine 5′-triphosphate (*ATP*) (5 mM). The level of IL-1β in the cell culture supernatant was measured 20 h later by enzyme-linked immunosorbent assay. Data are presented as mean + SEM of three independent experiments and compared using one-way ANOVA and Tukey's test; *** P < 0.001

Given the critical role of IL-1β in immunity and inflammation during infection [23], we investigated the effect of EgCF on IL-1β secretion by macrophages. Mouse peritoneal macrophages (PM) were primed with LPS in the presence or absence of EgCF for 4 h and then exposed to nigericin or ATP as the second signal to activate NLRP3 inflammasome for 20 h. ELISA results confirmed successful NLRP3 inflammasome activation, as demonstrated by a significant increase in mature IL-1β levels in the LPS plus NLRP3 agonists group compared to the control group. However, EgCF treatment significantly inhibited IL-1β release stimulated by LPS plus nigericin or LPS plus ATP (Fig. 1A). A similar reduction of IL-1β production was observed in THP-1 cells (Fig. 1B). These results demonstrated that EgCF could suppress the secretion of IL-1β by macrophages.

### EgCF promotes ROS levels in macrophages

**Fig. 2:**
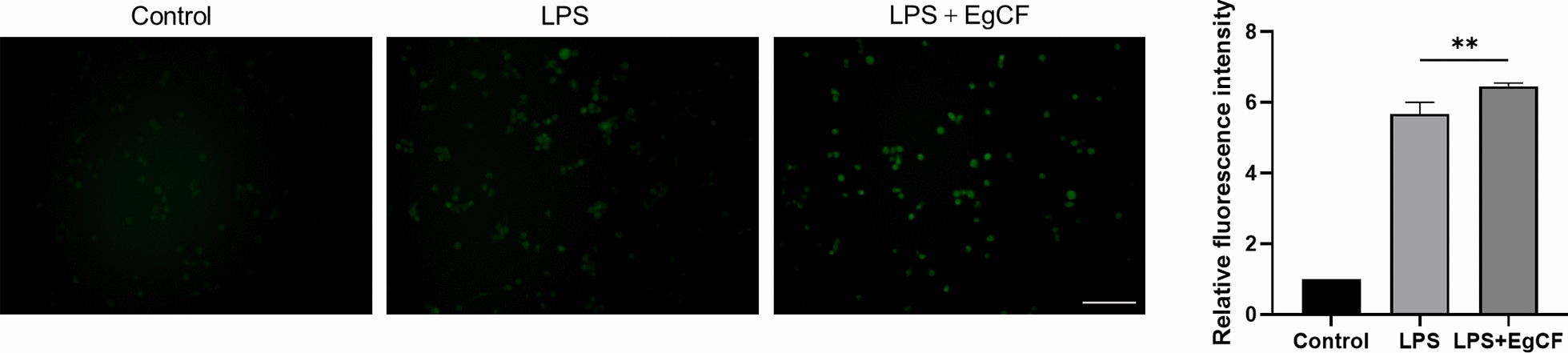
EgCF promotes reactive oxygen species (ROS) levels in macrophages. THP-1 cells were treated with EgCF (50% v/v) and LPS (1 μg/mL) for 4 h, and ROS levels were evaluated using a dichlorodihydrofluorescein diacetate probe. Bars indicate 50 μm. Data are presented as mean + SEM of three independent experiments and compared using one-way ANOVA and Tukey's test; ** P < 0.01. For other abbreviations, see Fig. 1

The production of ROS is considered an upstream signaling event for activating NLRP3 inflammasome [22]. Therefore, we examined the effect of *E. granulosus* on ROS levels, using a dichlorodihydrofluorescein diacetate probe. As shown in Fig. 2, EgCF could promote ROS production, indicating that the inhibitory effect of EgCF on IL-1β maturation is not caused by the inhibition of the second signal of NLRP3 inflammasome activation.

### EgCF inhibits NLRP3 transcription in macrophages

**Fig. 3:**
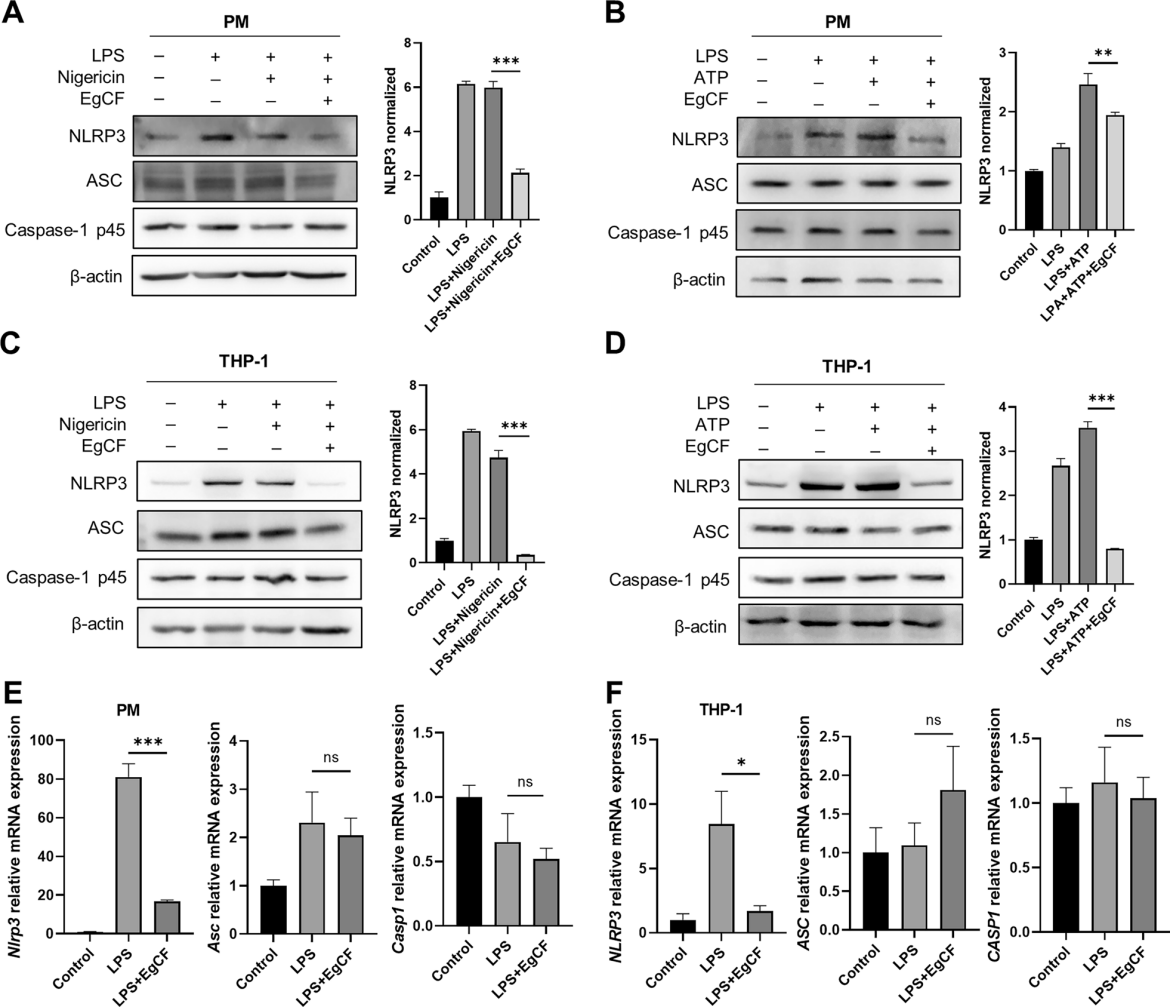
**A**–**F** EgCF inhibits NLRP3 transcription triggered by inflammasome inducer in macrophages. PM and THP-1 cells were primed with LPS in the presence or absence of EgCF and then exposed to nigericin or ATP. Protein levels of NLRP3, ASC, and caspase-1 p45 were measured by western blotting (**A**–**D**). *NLRP3*, *ASC*, and *CASP1* messenger RNA (*mRNA*) levels were assessed using quantitative real-time polymerase chain reaction (qRT-PCR) (**E**, **F**). Data are presented as mean + SEM of three independent experiments and compared using one-way ANOVA and Tukey’s test; * P < 0.05, ** P < 0.01, *** P < 0.001,* ns* not significant. For other abbreviations, see Fig. 1

Based on the ROS results, it was hypothesized that EgCF acts directly on the NLRP3 inflammasome complex instead of inhibiting the second signal of NLRP3 inflammasome activation. PM were primed with LPS and then stimulated with nigericin. The protein level of NLRP3 was found to be significantly decreased by EgCF, while ASC and caspase-1 p45 were unaffected (Fig. 3A). Similarly, it was noted that EgCF potently suppressed NLRP3 in response to ATP (Fig. 3B). In THP-1 cells, NLRP3 was consistently upregulated after LPS combined with nigericin or ATP stimulation, and this upregulation was inhibited by EgCF (Fig. 3C, D). NLRP3 transcription levels were analyzed by qRT-PCR, which revealed that LPS upregulated NLRP3 transcription over 4 h of stimulation. After treatment with EgCF, transcription levels of *NLRP3*, but not *ASC* and *CASP1*, were significantly lowered in both PM and THP-1 cells (Fig. 3E, F). Furthermore, we performed kinetic studies to understand the differences between messenger RNA (mRNA) and protein expression of NLRP3 levels (Additional file 2: Figure S1). These results showed that the *NLRP3* transcription level was significantly lowered from 2 h post-EgCF treatment in THP-1 cells; the NLRP3 protein level was significantly decreased by EgCF from 4 h post-EgCF treatment and was more potently suppressed at 16 h. These findings suggest that EgCF suppresses the transcription of NLRP3 in both mouse and human macrophages.

### EgCF inhibits LPS-induced IL-1β transcription

**Fig. 4:**
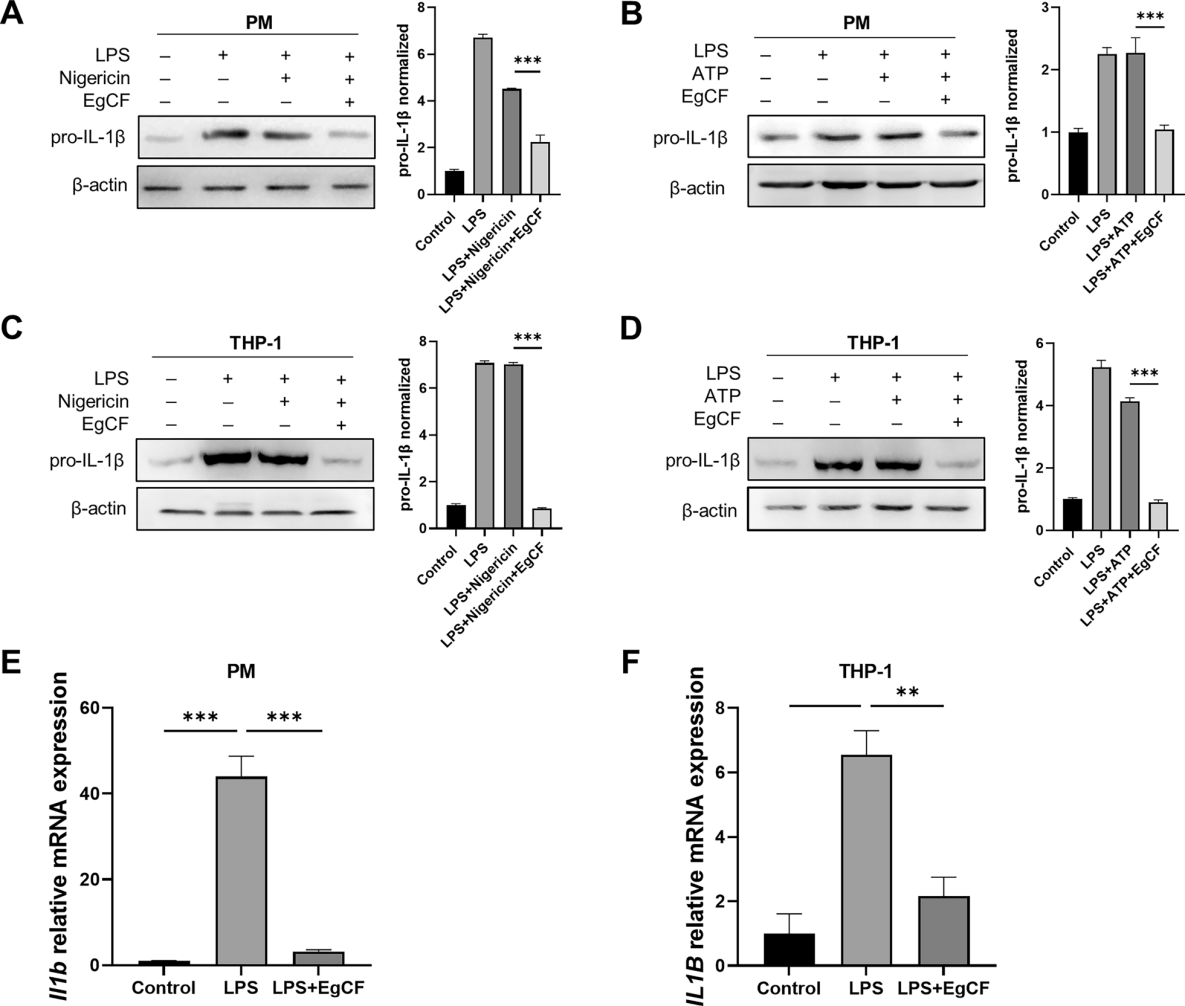
**A**–**F** EgCF inhibits the transcription of IL-1β triggered by inflammasome inducers in macrophages. PM and THP-1 cells were primed with LPS in the presence or absence of EgCF and then stimulated with nigericin or ATP. Pro-IL-1β protein levels were detected by western blotting (**A**–**D**). IL-1β mRNA levels were detected by qRT-PCR (**E**, **F**). Data are presented as mean + SEM of three independent experiments and compared using one-way ANOVA and Tukey’s test; ** *P* < 0.01, *** *P* < 0.001. For other abbreviations, see Figs. 1 and  3

IL-1β is produced in the form of pro-IL-1β, which is converted to mature IL-1β through post-translational processing [22]. To evaluate the effect of EgCF on the synthesis of pro-IL-1β, we conducted experiments on PM stimulated with LPS or LPS plus nigericin. Results demonstrated that the treatment of PM with LPS or LPS plus nigericin led to an increase in the level of pro-IL-1β. However, EgCF treatment suppressed the synthesis of pro-IL-1β (Fig. 4A). Additionally, we observed that the elevated expression of pro-IL-1β induced by LPS plus ATP was inhibited by EgCF (Fig. 4B). Likewise, EgCF was able to impede the expression of pro-IL-1β induced by LPS plus nigericin or LPS plus ATP in THP-1 cells (Fig. 4C, D). Furthermore, EgCF treatment significantly reduced the mRNA level of IL-1β induced by LPS (Fig. 4E, F), suggesting that EgCF acts as an inhibitor of pro-IL-1β synthesis in macrophages.

### EgCF promotes the enrichment of H3K27me3 at the promoter of NLRP3 and IL-1β

**Fig. 5:**
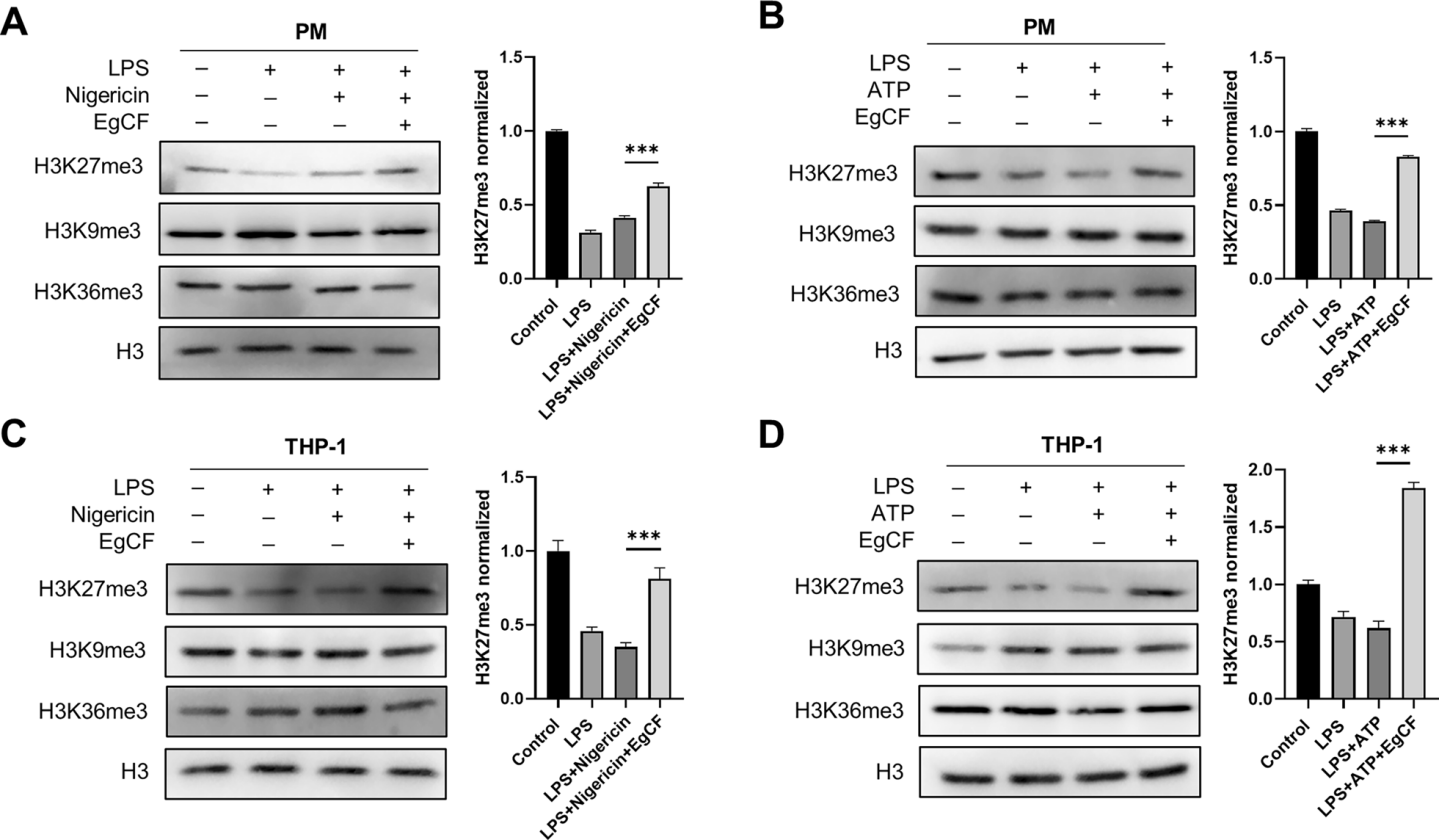
**A**–**D** EgCF promotes the global level of trimethylated histone H3 lysine 27 (*H3K27me3*). PM and THP-1 cells were primed with LPS in the presence or absence of EgCF and then exposed to nigericin or ATP. Cell lysates were immunoblotted for H3K9me3, H3K27me3, and H3K36me3. Data are presented as mean + SEM of three independent experiments and compared using one-way ANOVA and Tukey’s test; *** P < 0.001. For other abbreviations, see Figs. 1 and  3

Pathogens have developed various epigenetic strategies to survive within host cells [24]. Methylation of H3K9 and H3K27 is associated with repression of transcription, whereas methylation of H3K4 and H3K36 is commonly linked to active transcription [24, 25]. Our previous study showed that EgCF could inhibit the enrichment of H3K4me3 at TNF-α and IL-6 promoters. Here, we further detected the global methylation levels of H3K9, H3K27, and H3K36 in macrophages stimulated with LPS plus nigericin or ATP by western blotting to evaluate the effect of EgCF on histone modifications. The results showed that EgCF specifically enhanced H3K27me3 levels in PM and THP-1 cells, whereas no consistent and apparent changes were found for the levels of H3K9me3 and H3K36me3 (Fig. 5A–D). These data indicated that EgCF regulates H3K27me3 levels, thus influencing host gene expression.

**Fig. 6:**
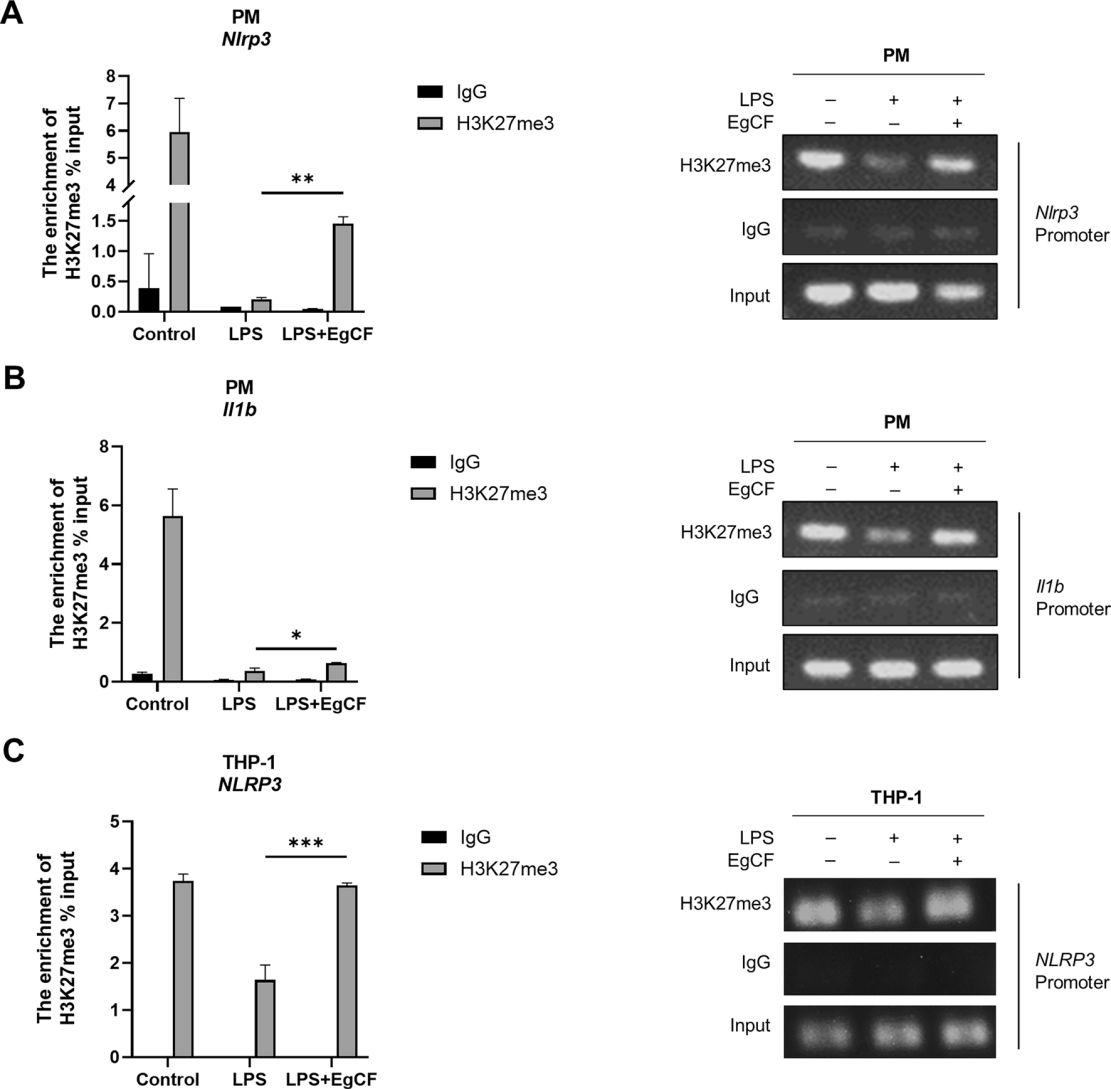
**A**–**C** EgCF induces higher H3K27me3 levels at the promoters of NLRP3 and IL-1β. Chromatin immunoprecipitation was performed in PM and THP-1 cells treated with EgCF and LPS for 4 h. qRT-PCR was used to detect the enrichment of H3K27me3 modification at NLRP3 and IL-1β promoters (left). The amplified products were visualized by agarose gel electrophoresis (right). Data are presented as mean + SEM of three replicates, representing one of three independent experiments, and compared using one-way ANOVA and Tukey’s test; * P < 0.05, ** *P* < 0.01, *** P < 0.001. For abbreviations, see Figs. 1,  3 and 5

To investigate whether H3K27me3 modification is involved in the EgCF-mediated suppression of NLRP3 transcription, we performed a ChIP-PCR assay to detect the enrichment of H3K27me3 at the NLRP3 promoter. Our findings showed a significant decrease in the H3K27me3 signal at the NLRP3 promoter in PM stimulated with LPS compared to control cells (Fig. 6A; Additional file 3: Figure S2). In contrast, the EgCF treatment effectively restored H3K27me3 levels (Fig. 6A). Similarly, we observed increased H3K27me3 levels at the IL-1β promoter in PM treated with EgCF (Fig. 6B). Furthermore, we also observed a considerable amount of suppressive H3K27me3 at the promoter of NLRP3 in THP-1 cells treated with EgCF (Fig. 6C). Our results demonstrated that EgCF treatment in macrophages induced the enrichment of H3K27me3 at the promoters of NLRP3 and IL-1β.

### EgCF inhibits LPS-induced demethylase KDM6B expression in macrophages

**Fig. 7:**
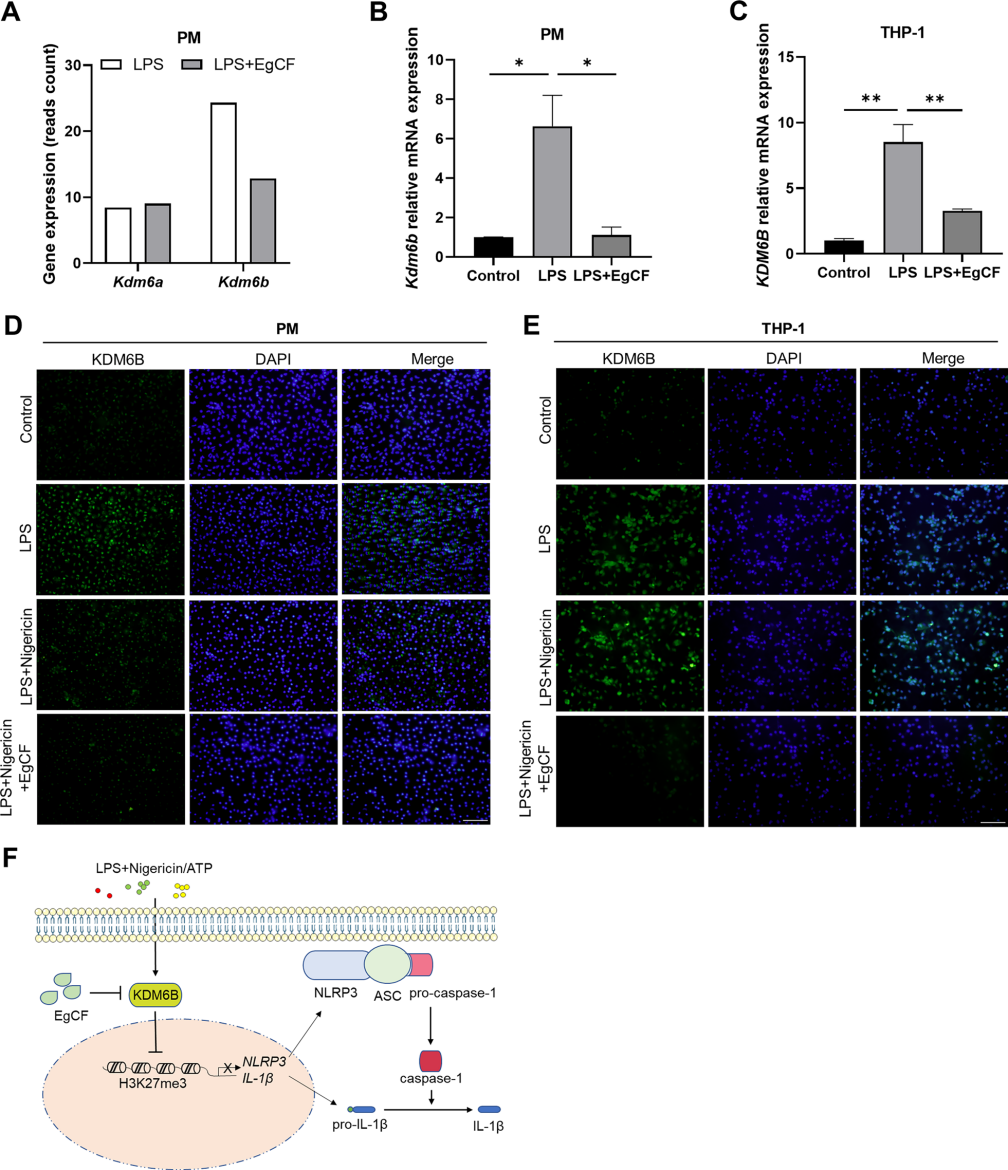
**A**–**F** EgCF inhibits the expression of KDM6B in macrophages induced by LPS. The expression of H3K27me3 demethylases was measured by RNA-sequencing (**A**). The mRNA level of KDM6B in PM and THP-1 cells was detected by qRT-PCR (**B**, **C**). Protein levels of KDM6B were determined by using immunofluorescence staining (**D**, **E**). Bars indicate 50 μm. Data are presented as mean + SEM of three independent experiments and compared using one-way ANOVA and Tukey's test; * *P* < 0.05, ** P < 0.01.* DAPI* 4′6-Diamidino-2-phenylindole; for other abbreviations, see Figs. 1,  3 and 5

KDM6A and KDM6B are demethylases critically responsible for H3K27me3 in mammals [26]. Through RNA-sequencing data analysis of PM treated with LPS and EgCF, we observed that expression of KDM6B mRNA was inhibited by EgCF, whereas KDM6A mRNA expression was unaffected (Fig. 7A). This finding led us to hypothesize that EgCF enhances the H3K27me3 level by downregulating KDM6B. We performed qRT-PCR with PM and THP-1 cells treated with EgCF to test this hypothesis, and found that EgCF treatment significantly decreased LPS-induced KDM6B transcription (Fig. 7B, C). Moreover, fluorescence staining revealed that the KDM6B protein level increased with NLRP3 inflammasome activation in PM and could be suppressed by EgCF (Fig. 7D). KDM6B expression was also consistently reduced in THP-1 cells (Fig. 7E). These data indicated that EgCF could inhibit LPS-induced KDM6B expression in macrophages.

## Discussion

CE is caused by *E. granulosus* sensu lato. This parasite has developed effective strategies to evade its host immune system [5]. EgCF, which is a mixture of excretory and secretory products derived from protoscoleces and the germinal layer, facilitates immune evasion by exerting cytotoxic and modulatory effects on host immune cells [6, 27]. Proteomic analysis has indicated that the hydatid walls are permeable, allowing for a high exchange rate of proteins between the metacestode and the surrounding tissues of the host [28–30]. For instance, EgCF could inhibit the inflammatory response of human and mouse macrophages by blocking TRAF6 and NF-κB downstream activation [31]. In this study, we established an NLRP3 activation model in macrophages by exposing them to LPS, nigericin, and ATP. We found that EgCF treatment could inhibit the inflammatory response by suppressing the expression of KDM6B, which increased the enrichment of H3K27me3 modification to NLRP3 and IL-1β. As a result, the transcription of NLRP3 and IL-1β, as well as the secretion of mature IL-1β, was inhibited in both mouse peritoneal macrophages and THP-1 cells (Fig. 7F).

The activation of NLRP3 results in the recruitment of the adaptor protein ASC and the cysteine protease caspase-1, forming an inflammasome that triggers the cleavage of pro-IL-1β into its mature secreted forms [22]. We first hypothesized that EgCF inhibits IL-1β expression by suppressing NLRP3 inflammasome cleavage activity. To test this, we measured intracellular ROS, one of the second signals of NLRP3 inflammasome activation. Surprisingly, we found that EgCF promoted the production of ROS instead of inhibiting it. This finding agrees with that of a previous study that utilized flow cytometry and demonstrated the ability of EgCF to induce oxidative damage in macrophages and increase ROS levels [32]. Our results suggest that the cleavage of NLRP3 inflammasome does not significantly impact the reduction of IL-1β secretion. Instead, we discovered that EgCF could suppress the transcription of NLRP3. Additionally, EgCF interferes with IL-1β transcription and translation, as confirmed by qRT-PCR and western blotting. This raises questions about how EgCF inhibits NLRP3 and IL-1β gene transcription. Interestingly, similar results have been observed in primary human neutrophils, where *T. gondii* inhibits NLRP3 and IL-1β production induced by LPS or LPS plus ATP [11].

Histone modifications can alter gene expression without changing the chromosomes [33]. H3K27me3 is a common histone modification that represses transcription [34]. Previous research showed that GSKJ4 could impede the inflammatory response by inhibiting KDM6B and augmenting the NLRP3 transcriptional suppression in colitis [35]. Additionally, increased expression of KDM6B has been found to lead to reduced H3K27me3 modification at the promoters of the IL-1β genes, resulting in the secretion of IL-1β in macrophages [20, 36]. Based on these findings, we hypothesized that EgCF could inhibit NLRP3 and IL-1β expression by increasing the level of H3K27me3 modification. Our experiments revealed that EgCF could increase the levels of global H3K27me3 compared to LPS-primed macrophages. We also conducted a ChIP assay and found that EgCF enhanced the enrichment of H3K27me3 at the promoters of NLRP3 and IL-1β. KDM6B is a demethylase that regulates H3K27me3 [17], and qRT-PCR and fluorescence immunoassay demonstrated that EgCF decreased both the mRNA and protein levels of KDM6B. To our knowledge, this study is the first to propose a model where tapeworms repress the expression of NLRP3 and IL-1β, primarily through the transcriptional suppression of histone demethylase.

EgCF is composed of proteins, chemical elements, biochemical metabolites, and free amino acids [6]. Our previous [37] study showed that glycomolecules in EgCF could interfere with the TLR4-mediated inflammatory responses via c-Raf in dendritic cells. However, which component is critical for EgCF–macrophage immune modulation and KDM6B regulation needs to be further explored. Subjecting EgCF to heat denaturation of its proteins or periodate treatment for the modification of its carbohydrates may provide clues to understanding possible factors that drive the immune escape [37].

In summary, our findings demonstrate that EgCF can inhibit KDM6B expression and the demethylation of H3K27me3, leading to the inhibition of NLRP3 and IL-1β transcription. This negative regulation ultimately results in the inhibition of the inflammatory response. These findings offer new insights for a better understanding of the immune escape induced by *E. granulosus*.

### Supplementary Information

**Additional file 1:** Original western blots probed with the indicated antibodies in PM and THP-1 cells, as shown in **Figures 3**–**5** and **S1**.

**Additional file 2: Figure S1. **Kinetic studies of NLRP3 levels. THP-1 cells were primed with LPS in the presence or absence of EgCF. **A** *NLRP3 *mRNA levels were assessed using qRT-PCR. **B** Protein levels of NLRP3 were measured by western blotting. Data are presented as mean + SEM of three independent experiments and compared using one-way ANOVA and Tukey’s test. * P < 0.05, ** *P* < 0.01, *** P < 0.001

**Additional file 3: Figure S2. **Raw electrophoresis images. **A** Enrichment of H3K27me3 modification at *Nlrp3* (left) and *Il1b* (right) promoters in mouse peritoneal macrophages.* M* Marker,* 1* input control,* 2* input LPS,* 3* input LPS + EgCF,* 4* H3K27me3 control,* 5* H3K27me3 LPS,* 6* H3K27me3 LPS + EgCF,* 7* IgG control,* 8* IgG LPS,* 9* IgG LPS + EgCF. **B** Enrichment of H3K27me3 modification at the *NLRP3* promoter in THP-1 cells.

## Data Availability

All of the data generated or analyzed during this study are included in this published article.
