# Supplementary material for: Echinococcus granulosus cyst fluid inhibits KDM6B-mediated demethylation of trimethylated histone H3 lysine 27 and interleukin-1β production in macrophages
Source: Parasit Vectors. 2023 Nov 16;16:422. doi: 10.1186/s13071-023-06041-3 (PMC10652454; doi:10.1186/s13071-023-06041-3)

Figure 3A: NLRP3 in PM


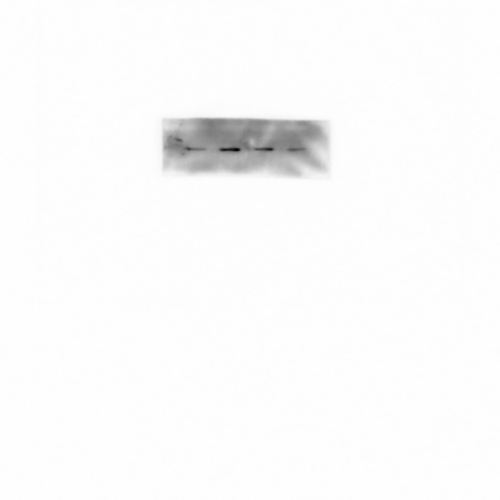


Figure 3A: ASC in PM


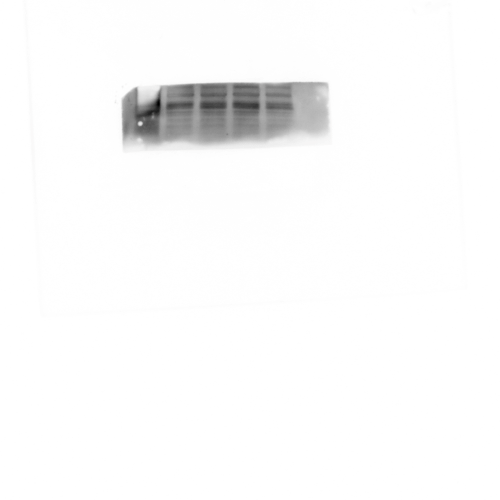


Figure 3A: Caspase-1 p45 in PM


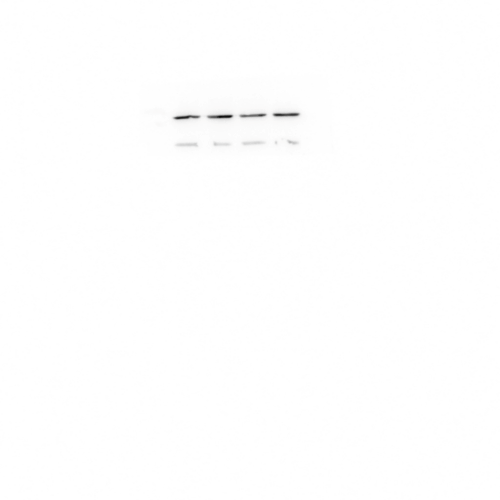


Figure 3A: β-actin in PM


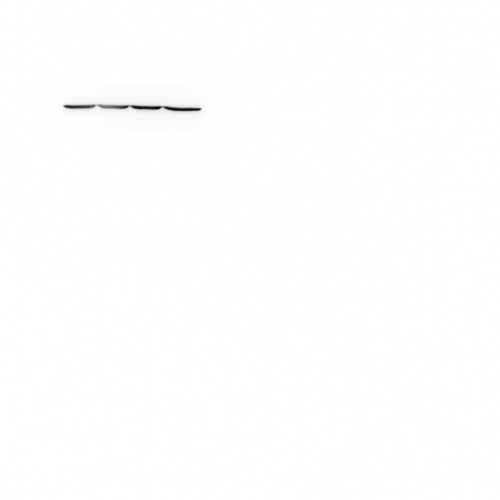


Figure 3B: NLRP3 in PM


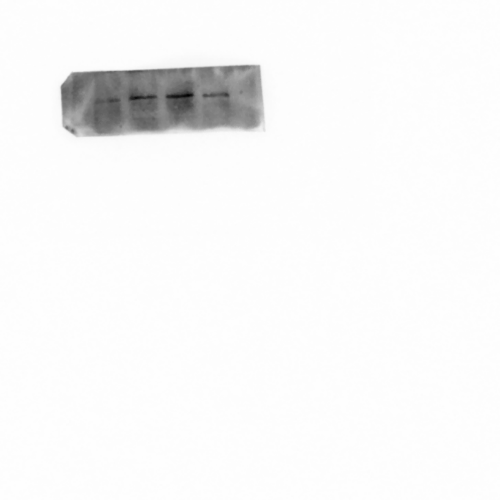


Figure 3B: ASC in PM


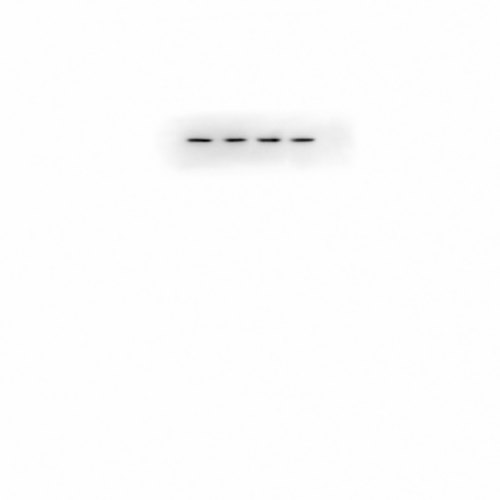


Figure 3B: Caspase-1 p45 in PM


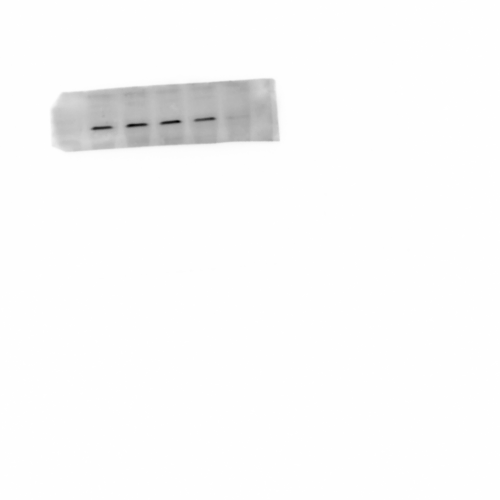


Figure 3B: β-actin in PM


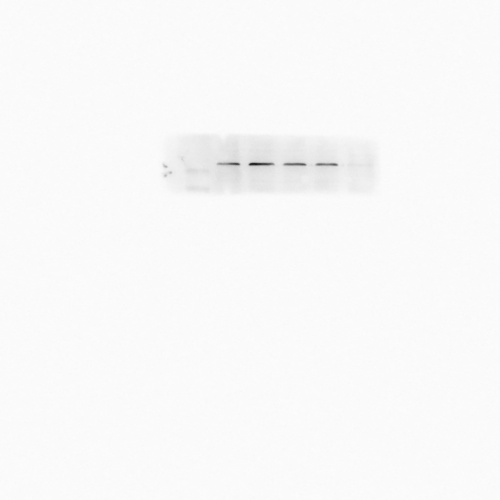


Figure 3C: NLRP3 in THP-1


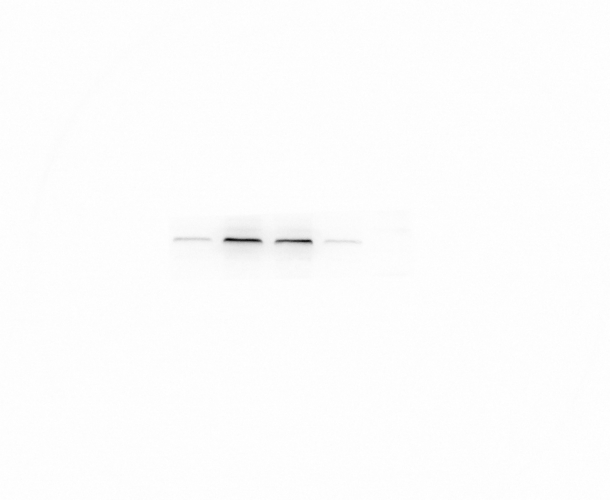


Figure 3C: ASC in THP-1


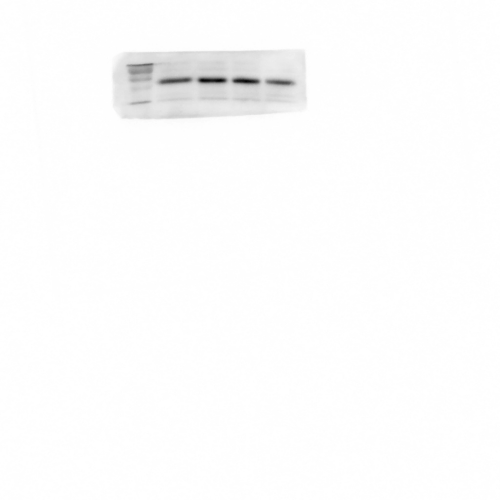


Figure 3C: Caspase-1 p45 in THP-1


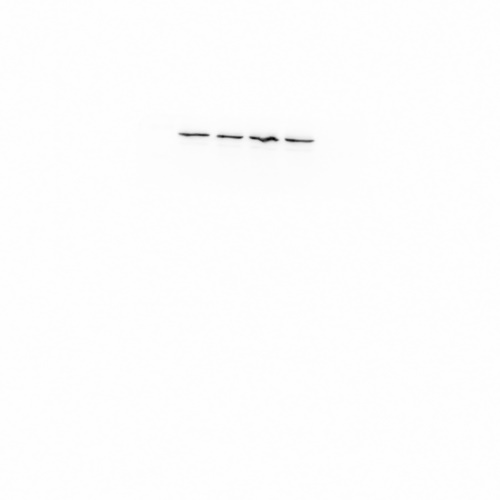


Figure 3C: β-actin in THP-1


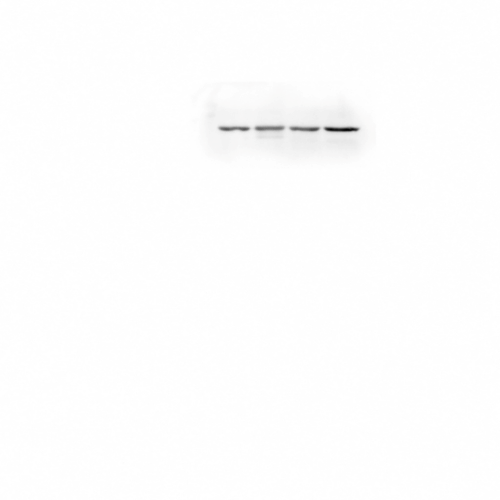


Figure 3D: NLRP3 in THP-1


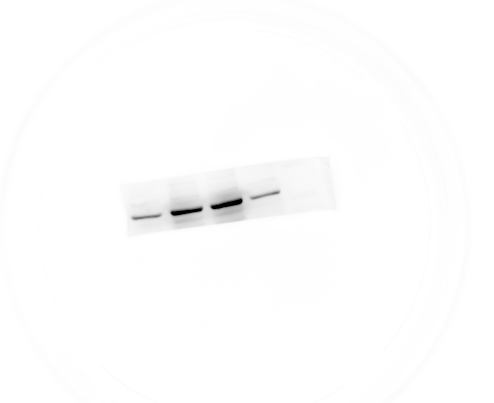


Figure 3D: ASC in THP-1


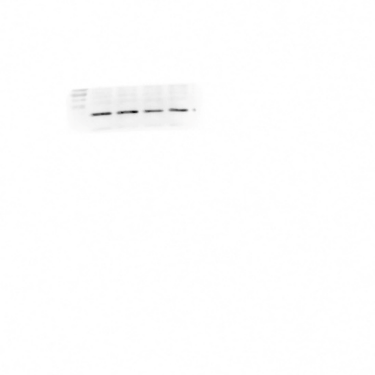


Figure 3D: Caspase-1 p45 in THP-1


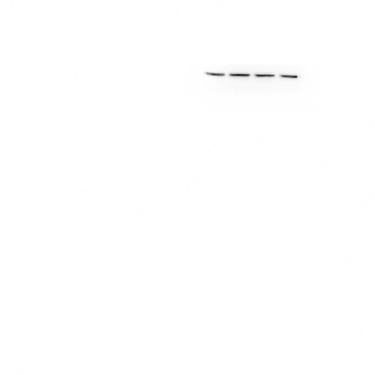


Figure 3D: β-actin in THP-1


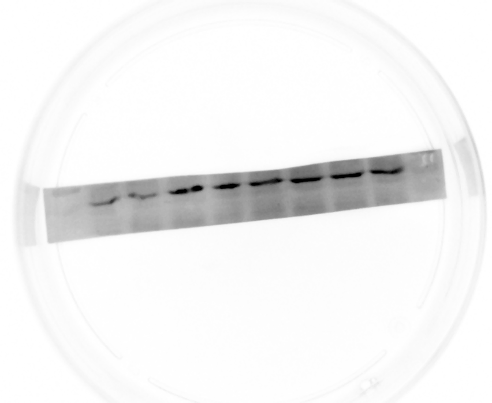


Figure 4A: pro-IL-1β in PM


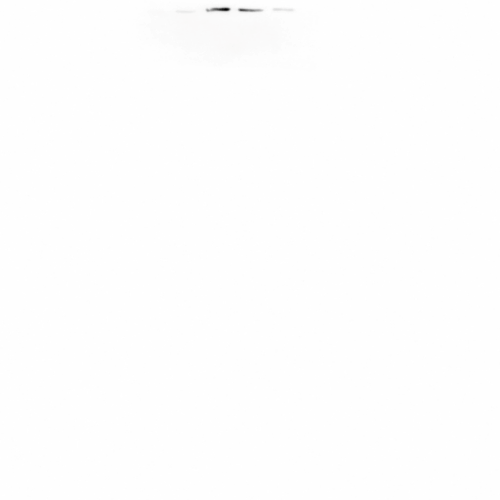


Figure 4A: β-actin in PM


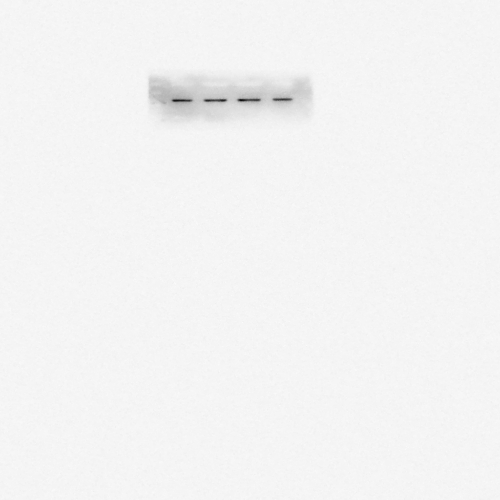


Figure 4B: pro-IL-1β in PM


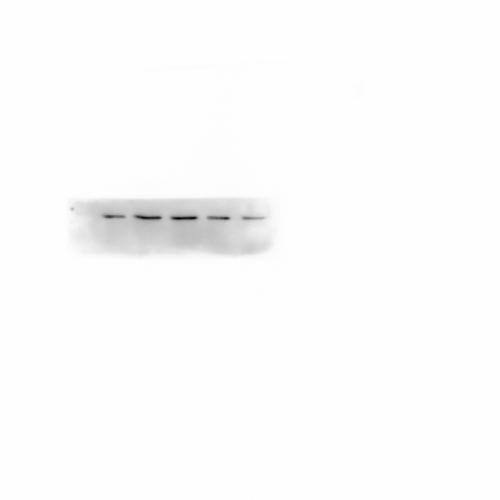


Figure 4B: β-actin in PM


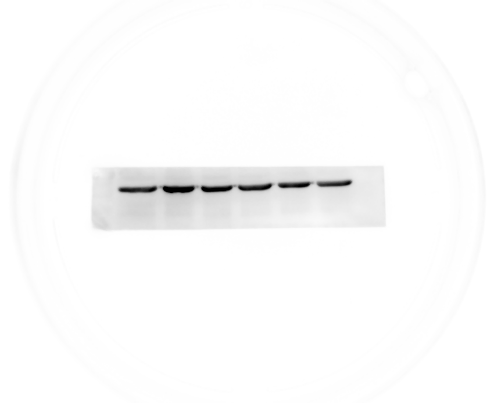


Figure 4C: pro-IL-1β in THP-1


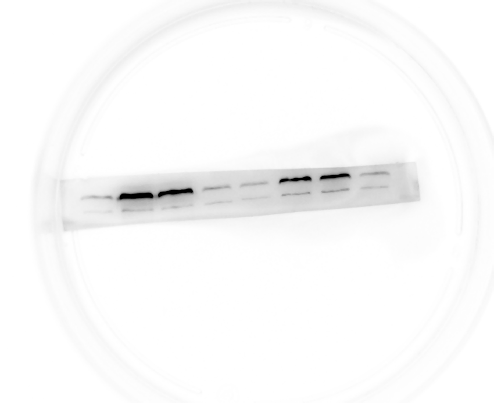


Figure 4C: β-actin in THP-1


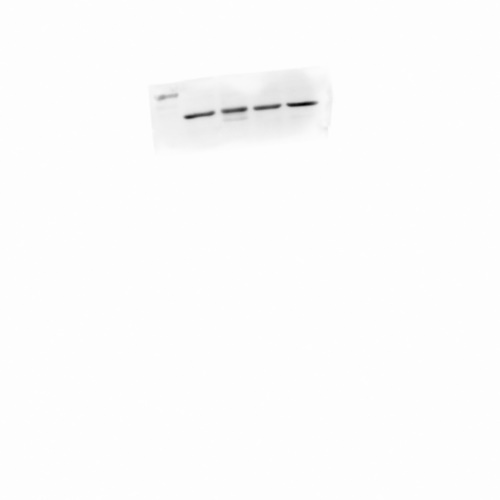


Figure 4D: pro-IL-1β in THP-1


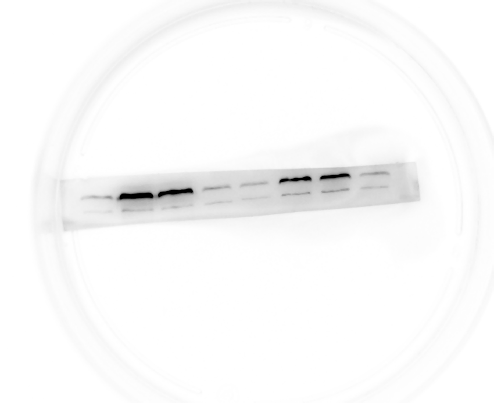


Figure 4D: β-actin in THP-1


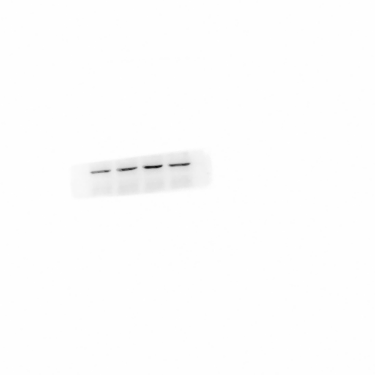


Figure 5A: H3K27me3 in PM


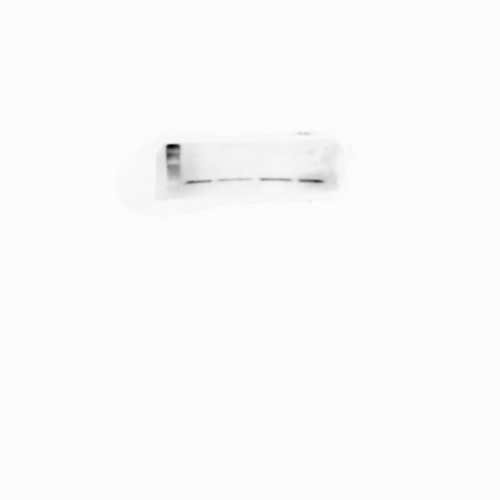


Figure 5A: H3K9me3 in PM


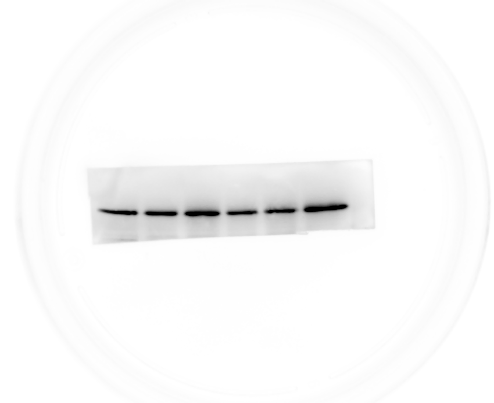


Figure 5A: H3K36me3 in PM


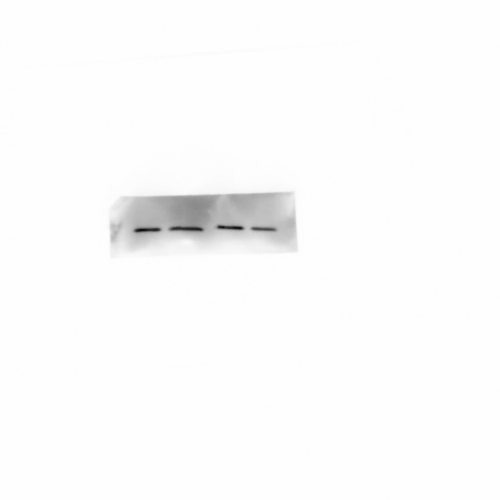


Figure 5A: H3 in PM


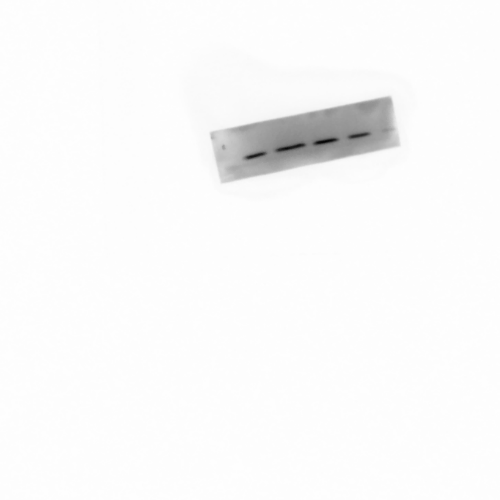


Figure 5B: H3K27me3 in PM


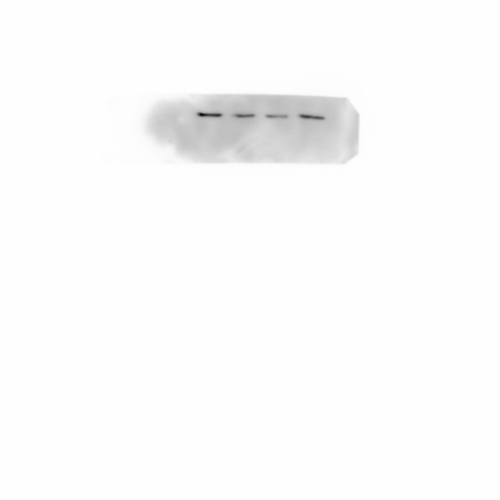


Figure 5B: H3K9me3 in PM


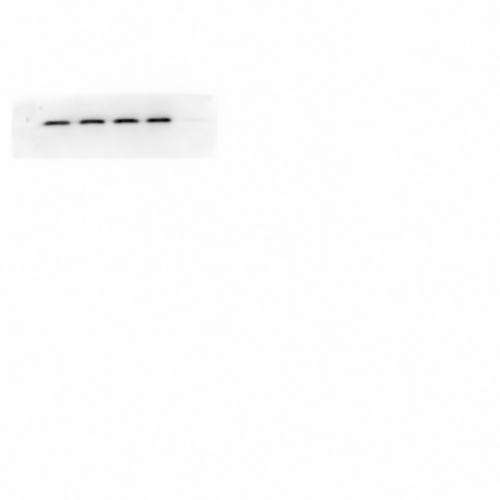


Figure 5B: H3K36me3 in PM


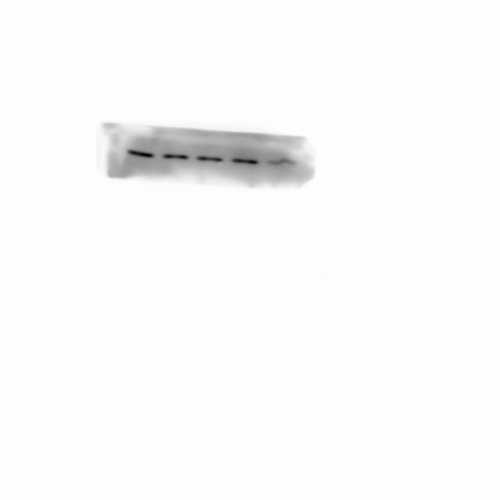


Figure 5B: H3 in PM


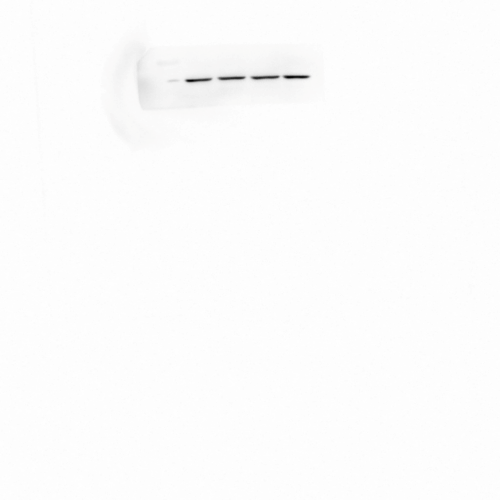


Figure 5C: H3K27me3 in THP-1


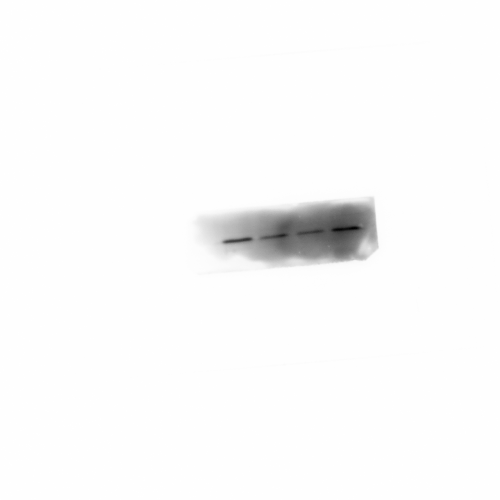


Figure 5C: H3K9me3 in THP-1


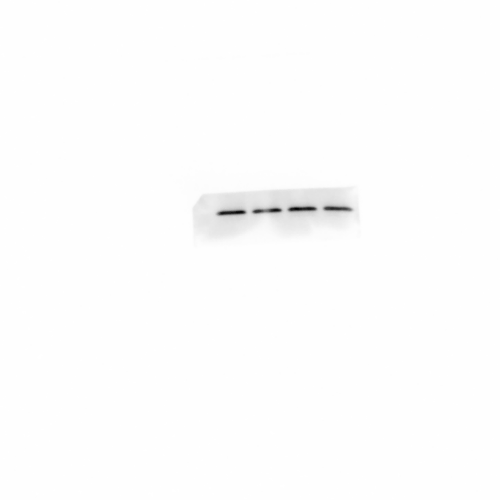


Figure 5C: H3K36me3 in THP-1


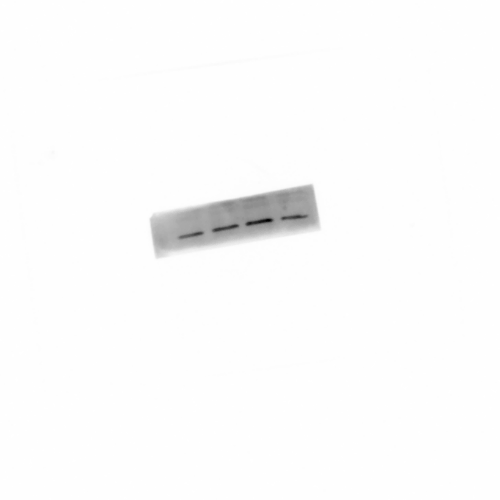


Figure 5C: H3 in THP-1


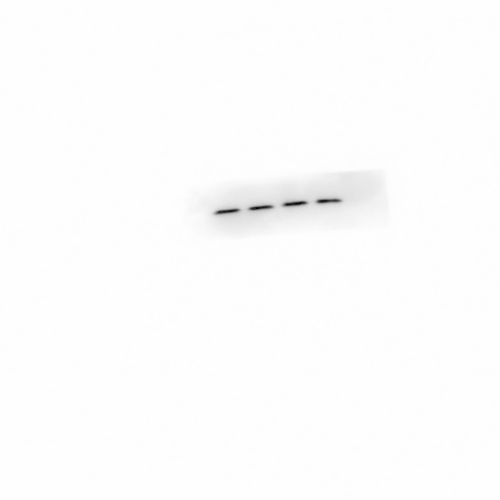


Figure 5D: H3K27me3 in THP-1


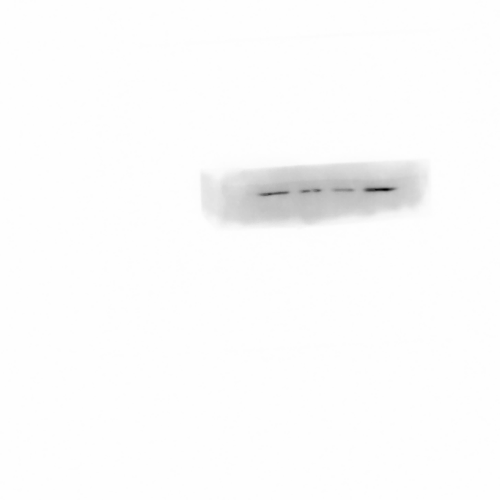


Figure 5D: H3K9me3 in THP-1


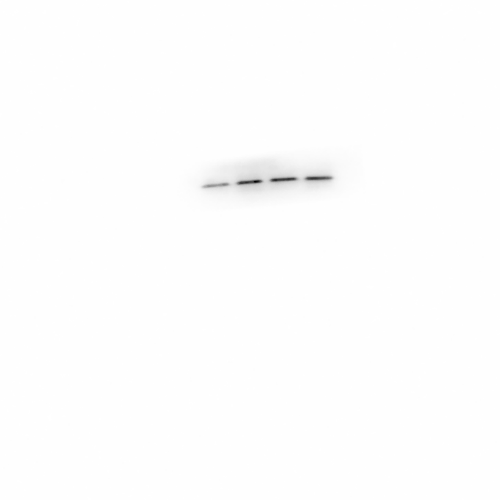


Figure 5D: H3K36me3 in THP-1


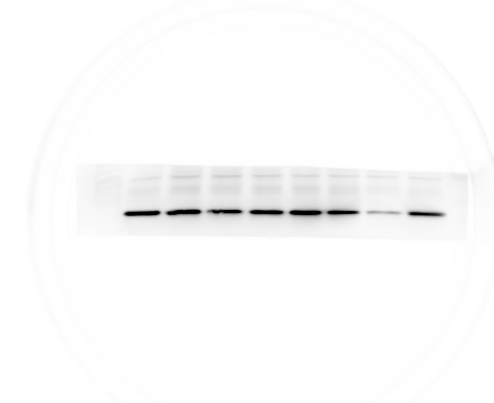


Figure 5D: H3 in THP-1


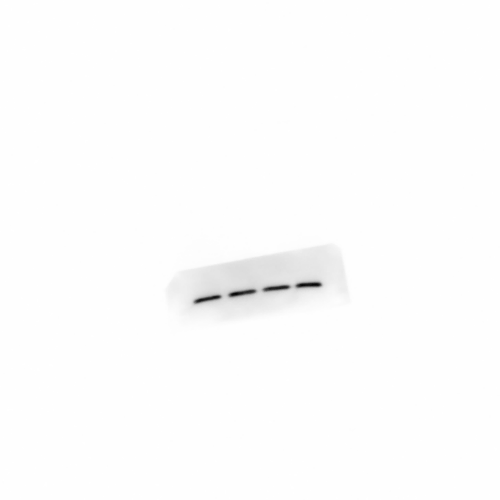


Figure S1B: NLRP3 in THP-1


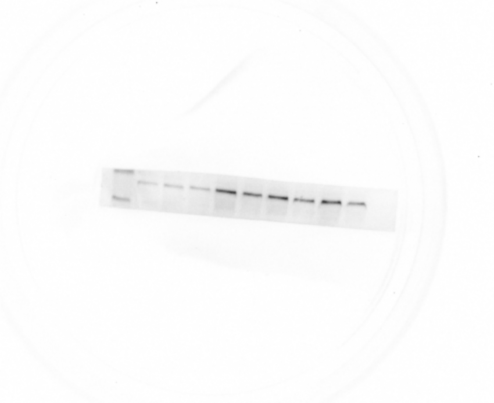


Figure S1B: β-actin in THP-1


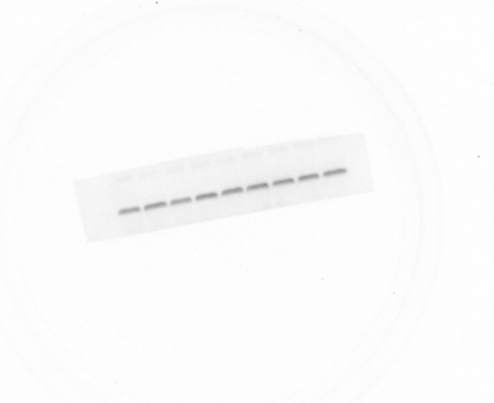

Supplement: Supplementary file 1 — Additional file 1: Original western blots probed with the indicated antibodies in PM and THP-1 cells, as shown in Figures 3–5 and S1. [file 13071_2023_6041_MOESM1_ESM.docx]
