# Supplementary figures and images for: Echinococcus granulosus cyst fluid inhibits KDM6B-mediated demethylation of trimethylated histone H3 lysine 27 and interleukin-1β production in macrophages
Source: Parasit Vectors. 2023 Nov 16;16:422. doi: 10.1186/s13071-023-06041-3 (PMC10652454; doi:10.1186/s13071-023-06041-3)

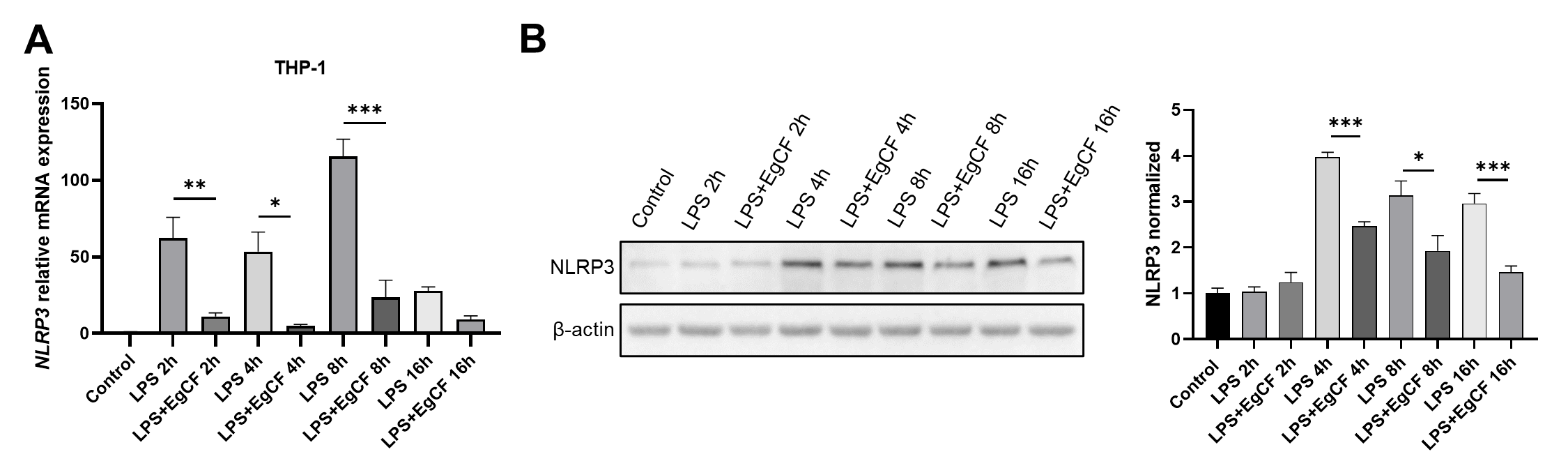

Supplement: Supplementary file 2 — Additional file 2: Figure S1. Kinetic studies of NLRP3 levels. THP-1 cells were primed with LPS in the presence or absence of EgCF. A NLRP3 mRNA levels were assessed using qRT-PCR. B Protein levels of NLRP3 were measured by western blotting. Data are presented as mean + SEM of three independent experiments and compared using one-way ANOVA and Tukey’s test. * P < 0.05, ** P < 0.01, *** P < 0.001 [file 13071_2023_6041_MOESM2_ESM.tif]

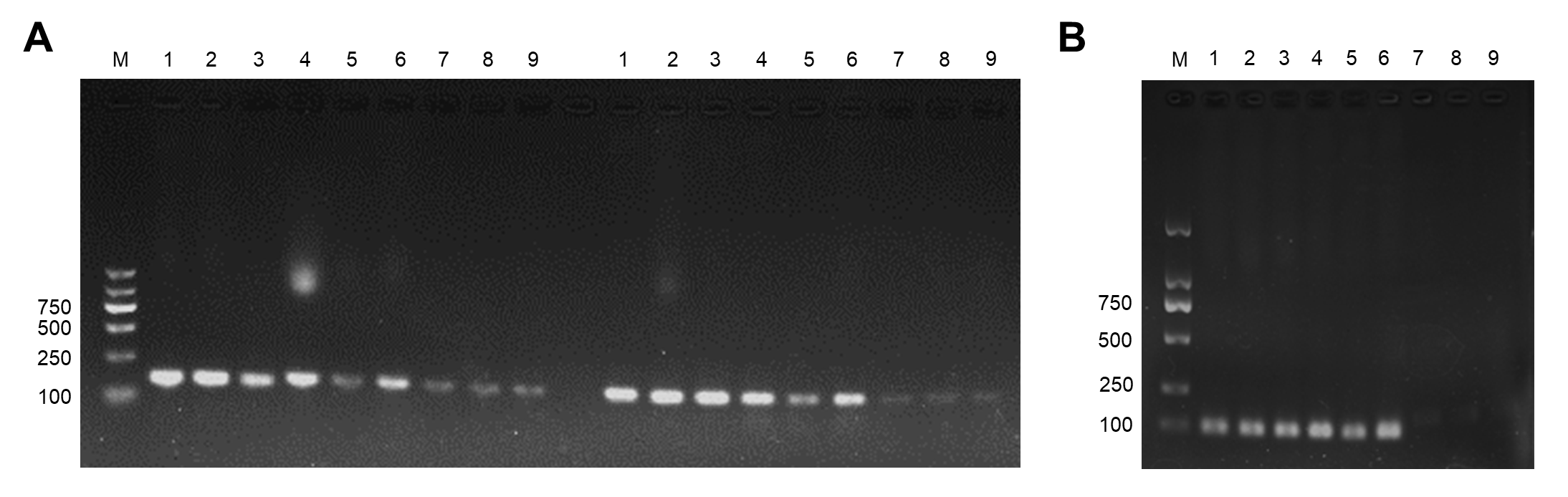

Supplement: Supplementary file 3 — Additional file 3: Figure S2. Raw electrophoresis images. A Enrichment of H3K27me3 modification at Nlrp3 (left) and Il1b (right) promoters in mouse peritoneal macrophages. M Marker, 1 input control, 2 input LPS, 3 input LPS + EgCF, 4 H3K27me3 control, 5 H3K27me3 LPS, 6 H3K27me3 LPS + EgCF, 7 IgG control, 8 IgG LPS, 9 IgG LPS + EgCF. B Enrichment of H3K27me3 modification at the NLRP3 promoter in THP-1 cells. [file 13071_2023_6041_MOESM3_ESM.tif]
